# Combined treatment of adenoid cystic carcinoma with cetuximab and IMRT plus C12 heavy ion boost: ACCEPT [ACC, Erbitux^® ^and particle therapy]

**DOI:** 10.1186/1471-2407-11-70

**Published:** 2011-02-15

**Authors:** Alexandra D Jensen, Anna Nikoghosyan, Axel Hinke, Jürgen Debus, Marc W Münter

**Affiliations:** 1 Dept. of Radiation Oncology INF 400 69120 Heidelberg Germany; 2 WiSP Wissenschaftlicher Service Pharma GmbH Karl-Benz-Str. 1 40764 Langenfeld Germany

## Abstract

**Background:**

Local control in adjuvant/definitive RT of adenoid cystic carcinoma (ACC) is largely dose-dependent leading to the establishment of particle therapy in this indication. However, even modern techniques leave space for improvement of local control by intensification of local treatment. Radiation sensitization by exploitation of high EGFR-expression in ACC with the EGFR receptor antibody cetuximab seems promising.

**Methods/design:**

The ACCEPT trial is a prospective, mono-centric, phase I/II trial evaluating toxicity (primary endpoint: acute and late effects) and efficacy (secondary endpoint: local control, distant control, disease-free survival, overall survival) of the combined treatment with IMRT/carbon ion boost and weekly cetuximab in 49 patients with histologically proven (≥R1-resected, inoperable or Pn+) ACC. Patients receive 18 GyE carbon ions (6 fractions) and 54 Gy IMRT (2.0 Gy/fraction) in combination with weekly cetuximab throughout radiotherapy.

**Discussion:**

The primary objective of ACCEPT is to evaluate toxicity and feasibility of cetuximab and particle therapy in adenoid cystic carcinoma.

**Trial Registration:**

Clinical Trial Identifier: NCT 01192087

EudraCT number: 2010 - 022425 - 15

## Background

Adenoid cystic carcinomas are rare tumors mostly of the head and neck accounting for approximately 10-15% of malignant salivary gland tumors [1]. They are characterised by a rather slow growth pattern but also perineural spread. Standard treatment so far consists of preferably complete surgical resection followed by adjuvant irradiation in case of close margins, perineural invasion, extensive primary tumor (T3, T4) or high-grade histology [2-5].

Local control in this disease could already be improved high-precision radiotherapy techniques such as stereotactic radiotherapy and/or intensity-modulated radiation therapy (IMRT), dose escalation, and high-LET RT. Treatment of large inoperable or residual tumors with IMRT resulted in increased local control as compared to conventional RT achieving progression-free survival rates of 38% at 3 years [6]. Neutron RT with a local control of 75% at 5 years, so far yielded the highest control rates in adenoid cystic carcinoma though unfortunately also accompanied by significant late toxicity [7,8].

Combined IMRT and C12 heavy ion boost are able to achieve similar or superior control rates as compared to neutron RT but without increase of late toxicity and subsequent morbidity. Recent results of our own group reported local control rates of 78% at 4 years [9] and 82% at 5 years respectively [10,11]. Our results are consistent with outcomes published by Mizoe et al in a smaller, more inhomogeneous patient cohort [12] leading to acceptance of our treatment regimen as a standard in Germany whenever available. Albeit progress has been made by the introduction of particle therapy in the treatment concept of adenoid cystic carcinoma, local control rates still leave room for improvement.

Novel paths have also been struck with radiochemotherapeutic approaches where various platin-containing regimen have been tested over the years but have not evolved beyond the phase II-stage or retrospective analyses [13-16].

Overall survival is unfortunately still limited by the occurence of distant metastases [6,17]. In recurrent or progressive metastatic disease, chemotherapy with cyclophosphamide, doxorubicine, and cisplatin ("CAP") is most commonly used as a therapeutic standard since the mid-80s [18-20] with response rates between 40 and 64% and remission rates of up to 24%. However, median duration of remission is generally only between 5-13 months [17,21].

More recently though, targeted therapies have raised expectations for the treatment of adenoid cystic carcinoma [22,23]. In view of high expression of EGF-receptors (up to 90%) in adenoid cystic carcinomas, therapy with the EGFR- antibody cetuximab seemed promising [22]. Despite the initial enthusiasm though, treatment results in recurrent and metastatatic adenoid cystic carcinoma have so far failed to impress: no objective response in recurrent or metastatic adenoid cystic carcinoma could be shown in any of the trials [24-26] although prolonged disease stabilization was observed in the reported series [25,26].

With the publication of the Bonner trial establishing radioimmunotherapy for squamous cell carcinoma of the head and neck (SCCHN) [27,28], the synergistic and radiosensitizing potential of cetuximab in combination with radiation therapy [29,30] was clinically proven. In view of the very mild and therefore attractive toxicity profile, cetuximab is a promising candidate to intensify local radiation therapy in adenoid cystic carcinoma. The ACCEPT trial was designed to evaluate toxicity and feasibility of the combination treatment (IMRT and carbon ion boost) with cetuximab.

## Methods/design

### Study design

The ACCEPT trial is a prospective, non-randomized phase II feasibility trial evaluating toxicity (any toxicity ≥ grade 3 CTCAE v. 4) of the combined treatment as primary endpoint.

### Study Characteristics

Combined IMRT and C12-heavy ion boost has previously been established as a treatment of choice in skull-base adenoid cystic carcinoma. In order to potentially further improve local control, the established treatment is complemented by the addition of cetuximab weekly. Therefore, the combination of IMRT (54 Gy in 2 Gy/fraction) and C12-boost (18 GyE in 3 GyE/fraction) and weekly Cetuximab will be tested as to toxicity profile and efficacy.

### Study objectives

To evaluate feasibility and toxicity of the combined treatment with IMRT/carbon ion boost and cetuximab are primary endpoints by assessing the incidence of any side effects with CTCAE ≥ grade 3. Secondary endpoints are local control, distant control, disease-free survival, and overall survival.

### Sample size/number of subjects

The trial design is based on the following assumptions:

• The experimental therapy is unacceptable if the true feasibility rate (:= 1 - withdrawal/dose limiting toxicity rate) is 70% or lower.

• The experimental therapy is considered promising if the true feasibility rate is 85% or more.

• Probability to accept the experimental therapy as well tolerated despite a true feasibility rate of <70%: 5% (type I error)

• Probability to reject the experimental therapy despite a true feasibility rate of >85%: 20% (type II error).

According to these parameters and using the variant out of the class of optimal two-stage designs by Simon [31] leading to the lowest required patient number, 23 pts evaluable for feasibility have to be recruited in the first stage. The combination will be rejected if seven or more of these patients fulfill the criterion of non-feasibility. Otherwise, further patients will be recruited in a second stage up to a total number of 49 subjects.

### Patient selection

#### Inclusion criteria

• Histologically proven adenoid-cystic carcinoma of the head and neck and Macroscopic or microscopic tumor rest (R1/R2) or

• Tumor stage >T3/T4 or

• perineural invasion and

• M0

• Written informed consent

• Age between 18 and 70 a

• Karnofsky-Index ≥ 70%

• adequate bone-marrow, liver, and kidney function

• effective contraception for patients in procreative age

#### Exclusion criteria

• prior RT or chemotherapy for tumors of the head and neck

• R0-resection

• M1 (metastases)

• prior immunotherapy

• signs of active infection

• other serious illnesses or medical conditions: therapy-refractory unstable heart disease, congestive heart failure NYHA °III and °IV; coagulopathies

• Other previous malignancy within the past 5 years except prior, adequately treated basal cell carcinoma of the skin or pre-invasive carcinoma of the cervix

• Significant neurological or psychiatric condition including dementia or seizures or other serious medical condition prohibiting the patient's participation in the trial by judgement of the investigators

• Legal incapacity or limited legal capacity

• Positive serum/urine β-HCG/pregnancy

• Drug abuse

### Radiotherapy

#### Immobilisation/planning examinations

Patients are immobilized using individual thermoplastic head masks with thermoplastic shoulder fixation. Planning examinations consist of a planning CT scan (3 mm slice thickness) with the patient positioned in the individual fixation device and contrast-enhanced MRI for 3D image correlation.

#### Target volumes/dose prescription

CTV1 (carbon ion boost) includes the macroscopic tumour/prior tumour bed with special focus on the R1-area as well as respective neural pathways to the base of skull (cave: perineural invasion and skip lesions). For tumours of the parotid gland, the whole former parotid area is also included in the CTV1, if possible the mandibular joint is kept outside the CTV1. PTV1 consists of a 3 mm margin around the CTV1 but does not extend into critical organs at risk (i.e. brain stem, spinal cord).

We prescribe a dose of 24 GyE carbon ions in 3 GyE/fraction (5 fractions per week) to the CTV1, we aim at covering the CTV1 with the 95% prescription isodose. Treatment is given at the HIT (Heidelberg ion therapy centre) after inverse treatment planning in active beam application (raster-scanning method). Daily image guidance consists of orthogonal x-ray controls in treatment position.

CTV2 includes CTV1 with safety margins along typical pathways of spread. Only ipsilateral nodal levels (II and III) are includes, however, in case the primary tumour is/was located at midline or crossing midline, we cover bilateral nodal levels II and III. In case there is a pathological lymph node involvement, additional nodal level will be covered as indicated. CTV2 also encompasses the complete surgical operational area. The CTV2 also takes account for set-up variations, hence corresponds to the PTV2 (CTV2 = PTV2). Should the primary tumour be located within the parotid gland, also the parotid duct needs to be within the CTV2.

50 Gy IMRT (inversely planned step-and-shoot or tomotherapy technique) in 25 fractions (5 fractions per week) are prescribed to the CTV2 (coverage at least with the 90% prescription isodose) taking into account doses applied by daily image guidance with MV-cone-beam CT.

### Immunotherapy

Cetuximab is administered as 400 mg/m2 body surface loading dose 7 days prior to RT-treatment start (d-7) after administration of anti-histamines (dimetindene) and corticosteroids (dexamethasone).

Weekly administrations of Cetuximab 250 mg/m2 body surface follow for the duration of radiotherapy from d1.

### Supportive therapy

Patients do not routinely receive prophylactic feeding tubes, however if they uncommonly experience significant weight loss we will of course offer feeding tube insertion or parenteral feeding.

### Treatment schedule/follow-up

#### Treatment schedule

**Figure 1:**
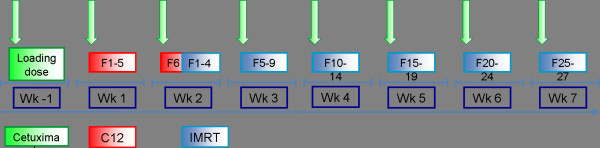
Trial flow-chart

After inclusion into the trial and the patient's written informed conset, the patient receives an individual positioning device (aquaplast mask incl. shoulder fixation) and RT treatment planning scans (CT/MRI). Subsequently, patients are administered Cetuximab loading dose on day -7 (400 mg/m² body surface). On day 1 (wk 1), the patient receives the first weekly Cetuximab (250 mg/m² body surface) as well as the first fraction of radiation therapy (figure 1).

First, the patient receives 6 fractions of C12 heavy ion boost to the GTV to a total dose of 18 GyE at 3 GyE per fraction. Subsequently, the patient receives intensitiy-modulated photon radiation therapy (IMRT) to the CTV to a dose of 54 Gy at 1.8 Gy per fraction (5 fractions per week). Overall, the total dose adds up to 72 GyE in 36 fractions.

Target localisation can either be stereotactically and/or under image guidance.

### Follow-up

**Figure 2:**
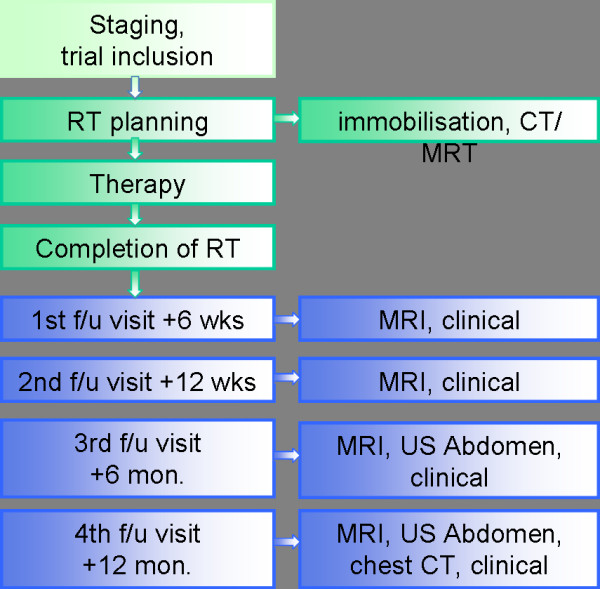
Trial schedule/follow-up

First follow-up examination including diagnostic, contrast-enhanced MRI will be carried out 6 weeks post completion of radiation treatment. Further controls including MRI are 3, 6, and 12 months thereafter, in 6 monthly intervals until 2 years post RT, then in yearly intervals (figure 2). Abdominal ultrasound will be carried out q6months, chest-CT q12months. At each follow-up appointment, patients receive a symptom-oriented clinical examination, also patients' performance state (Karnofsky-Index), therapy-associated side effects as well as potential intercurrent therapy is recorded.

Patients are also encouraged to undergo regular check-ups incl. full ENT clinical examinations in regular intervals.

### Trial duration

The trial fort he individual patient is completed after a 3-year follow-up period. Recruitement will p

### Assessment of efficacy

Assessment of efficacy will be carried out by evaluation of imaging studies (MRI) at each follow-up. If applicable (in case of initial macroscopic tumour), tumour response will be evaluated according to the RECIST-criteria [32].

### Proteomics and Genomics

For the proteomic examinations 30 mL venous blood will be collected from each subject prior to the first administration of IMP, at day 8 (before the first irradiation), at completion of the carbon ion boost (typically at day 14), at completion of radioimmunotherapy (typically day 54 of the treatment phase) and once at the 1st follow-up visit. Thus, the overall volume of blood samples used for Proteomic/Genomic investigations will be approximately 120 mL. Following parameters/pathways will be investigated:

• In order to predict the efficacy of the trimodal therapy blood will be collceted during therapy and follow-up to detect and correlate the levels of well known tumor- and angiogenesis markers (VEGF, TGF-Alpha, bFGF, IL8, k-ras, etc.) using Enzyme-Linked Immunosorbent Assay (ELISA). Further, platelet protein content (i.e. tumor angiogenesis growth factors and cytokines) will be analyzed using citrate blood samples and correlated with serum- and plasma- protein results.

• In order to perform the genomic analysis, patients' blood samples are collected as indicated and RNA, miRNA and DNA isolation will be performed. Based on an established platform, linear RNA-amplification, labelling and hybridization on human genome wide oligo-arrays (transcriptome analysis) are planned. DNA samples are used to identify potential chromosomal aberrations or epigenetic alterations that might predict treatment response. RNA and miRNA samples are further analyzed by real time quantitative RT-PCR to confirm microarray data and to test a subset of clinical predictors.

The determinations of proteomic and genomic parameters will be carried out at the Department of Radiation Oncology in Heidelberg.

No further genetic investigations on the blood collected during the study will be carried out!

### Trial organization/coordination

The ACCEPT trial has been designed by the Department of Radiation Oncology, University of Heidelberg, and is carried out at the Heidelberg Ion Therapy Centre (HIT). It is an investigator-initiated trial; the Department of Radiation Oncology is responsible for co-ordination, overall trial management, registration (clinicaltrials.gov Identifier: NCT 01192087); EudraCT registration (EudraCT number: 2010 - 022425 - 15), database management, quality assurance, monitoring, and reporting is carried out by WiSP Wissenschaftlicher Service Pharma GmbH, Langenberg, Germany.

### Investigators

Patients are recruited by the Department of Radiation Oncology, Heidelberg, Germany.

### Adverse events

Adverse and serious adverse events are recorded using NCI common toxicity criteria for adverse events (CTCAE v. 4). Acute radiation effects are defined as effects occurring within 90 days from beginning of radiotherapy. Late effects are defined as effects observed thereafter. Safety analysis is performed with respect to frequency of serious adverse events and adverse events stratified by organ system, severity, causality.

### Regular completion of the trial

Patient accrual is completed with inclusion of the last patient and should extend for approximately 2 years from trial initiation. Regular trial participation for each patient terminates 3 years post inclusion into the trial or the patient's death respectively.

### Discontinuation of treatment

• Patient wish

• Cetuximab treatment delay for more than 2 consecutive weeks,

• Occurrence of any grade 4 toxicities related to cetuximab,

• Occurrence of >/= grade 3 allergic/hypersensitivity reaction related to cetuximab.

• Medical condition necessitating treatment termination and withdrawal of the patient from the trial

• Pregnancy

• Lack of compliance

### Premature termination of the trial

The trial can be prematurely closed or suspended by the LKP in following cases:

• Medical or ethical reasons relevantly affecting the risk-benefit relationship,

• Difficulties in recruitment of subjects suggest unjustifiable prolongation of the study timeline,

• Previously unexpected adverse events (in respect of their nature, severity, duration or outcome) occur with unjustifiable frequency,

• Expected adverse events occur with an unexpectedly high incidence,

• Relevant superiority of patients in one treatment arm of a comparable clinical trial,

• Legal authorities' decision

The Ethics Committee (EC) and the competent regulatory authorities will be informed about premature closure of the trial. Furthermore, the Ethics Committee(s) and competent regulatory authorities themselves may decide to stop or suspend the trial.

If the trial is closed prematurely, the trial material such as completed, partially completed, and blank CRFs will be returned to the coordinating investigator.

All involved investigators have to be informed immediately about a cessation or suspension of the trial. The decision is binding on all investigators.

### Ethics, informed consent, and safety

The final protocol was approved by the University of Heidelberg Medical School ethics committee (AFmo-409/2010). The trial complies with the Helsinki Declaration in its recent German version, the Medical Association's professional code of conduct, principles of Good Clinical Practice (GCP) guidelines and the Federal Data Protection Act. It will be carried out in keeping with local legal and regulatory requirements. It is also subject to authorization by the German radiation protection authority (Bundesamt für Strahlenschutz: = BfS) and Paul-Ehrlich Institute (: = PEI). Medical confidentiality and Federal Data Protection Act will be followed. Written informed consent is obtained from each patient in oral and written form.

## Discussion

With the introduction of novel radiotherapy techniques like IMRT and particle therapy (neutron and carbon ion therapy), progress has been made toward higher local control rates in adenoid cystic carcinoma over the last decade.

Compared to neutron RT, carbon ion therapy has only mild side effects and since the opening of a new dedicated particle therapy facility (HIT) is now permanently available in Germany. However, we believe treatment outcome in adenoid cystic carcinoma still leaves room for improvement. While comparatively chemo-resistant, radiochemotherapy has not gained establishment as a standard approach in the treatment of adenoid cystic carcinoma so far. Since the introduction of the combined radiation and EGFR-inhibition as a standard concept for SCCHN though, targeted therapies such as the EGFR receptor antibody cetuximab have also gained interes. In view of the high expression of EGFR expression in ACC, the combination treatment of IMRT plus carbon ion boost and cetuximab promises synergistiv effects of radiation and EGFR inhibition and hence improvement of local control at the cost of only mild toxicity. The ACCEPT trial was designed to evaluate toxicity and efficacy of the combined treatment approach in a prospective phase I/II trial. To our knowledge, this is the first phase I/II trial combining particle therapy and targeted therapies.

## Competing interests

Prof. Dr. Dr. Debus is a member of the Merck KGaA advisory board, the other authors declare that they have no competing interests.

## Authors' contributions

ADJ, MWM, AH, and JD developed the study protocol and planned the trial. AH is responsible for statistical considerations/basis of the trial. ADJ, AN, MWM are responsible for conducting and co-ordination of the trial as well as patient recruitement. All authors read and approved the final manuscript

## Pre-publication history

The pre-publication history for this paper can be accessed here:

http://www.biomedcentral.com/1471-2407/11/70/prepub
